# Posterolateral Complex Reconstruction With Distal Femoral Varus Opening-Wedge Osteotomy for Unstable Neglected Multiligamentous Knee Injury With Valgus Malalignment

**DOI:** 10.5435/JAAOSGlobal-D-21-00102

**Published:** 2021-08-10

**Authors:** Hung-Kai Liao, Cheng-Pang Yang, Alvin Chao-Yu Chen, Yi-Sheng Chan

**Affiliations:** From the Department of Orthopedic Surgery, Chang Gung Memorial Hospital, Linkou Taoyuan and National Yang-Ming University Faculty of Medicine (Dr. Liao); the Department of Orthopedic Surgery, Division of Sports Medicine Chang Gung Memorial Hospital and Chang Gung University College of Medicine (Dr. Yang, Dr. Chao-Yu Chen, and Dr. Chan); and the Bone and Joint Research Center, Chang Gung Memorial Hospital, Linkou, Republic of China (Dr. Liao, Dr. Yang, Dr. Chao-Yu Chen, and Dr. Chan).

## Abstract

We presented a case of a 25-year-old woman with early posttraumatic degenerative change to the articular cartilage accompanied with valgus malalignment despite receiving anterior cruciate ligament reconstruction after a multiligamentous injury sustained 2 years earlier. Rapid deteriorating valgus malalignment may result from chronic instability and intra-articular bone loss. Simultaneous distal femoral varus osteotomy and posterolateral complex reconstruction were performed during a single surgery. Six months after the surgery, the patient could walk briskly and climb stairs without any discomfort. Salvage procedures and biological reconstruction could be the primary choice for young patients to recover their knee function while avoiding joint replacement.

Motor vehicle collisions and sport-related injuries are the two most common causes of multiligamentous knee injuries (MLKIs).^1,2^ MLKI is defined as the disruption of at least two of the four major stabilizing ligaments of the knee. In acute multiligament knee injury patients, detailed evaluation with physical examination, MRI, stress radiographs, and angiography are necessary if the ankle-brachial index is less than 0.9.^3^ Any missed ligament injury will result in incomplete preoperative planning, and associated lesions can compromise the newly reconstructed ligament because of residual instability. Furthermore, failure to completely address all contributing factors to knee instability will change the knee kinematics.^3^ It is also well known that chronic valgus instability and valgus malalignment cannot be controlled by ligament reconstruction alone.^4^ Reconstructing the ligaments and obtaining the correct mechanical alignment are advised for repairing MLKIs, especially in young patients. Although there are several treatment choices for osteoarthritis, arthroplasty is not suitable for osteoarthritis in young patients.

Much of the existing literature has focused on the evaluation and treatment choices for chronic posterolateral complex (PLC) deficiency in varus knee deformities.^5-7^ No case report has discussed a coexisting valgus deformity with PLC insufficiency. Given the rarity of these injuries and the lack of literature regarding preoperative planning, surgical reconstruction, and postoperative rehabilitation protocols, we present this case. We obtained the patient's informed written consent for publication of this case report.

## Case Report

This case describes a 25-year-old woman who was struck by a motorcycle 2 years ago. She decided to undergo anterior cruciate ligament reconstruction with artificial ligament augmentation and a reconstruction system instead of an autograft to avoid donor site morbidity. However, progressive right knee pain and the feeling of instability remained. After enduring it for 15 months, she came to sport medicine outpatient department for help. The degree of deformity and the frequency of swelling recurrences progressively increased, and she had been in extreme pain in recent months; thus, she came to our outpatient department for help.

### Physical Examinations

Right lower extremity revealed genu valgum deformity with apparent posterior sagging of the tibia, whereas the range of motion of the knee was complete. The posteriorly locked tibia impeded the performance of the anterior drawer test. The posterior drawer test and the posterolateral drawer test were both 3+. The varus stress test was 3+, and the valgus stress test was 2+. The McMurray test was negative, and the neurovascular test was intact without a presentation of footdrop.

### Imaging Evaluation

**Figure 1:**
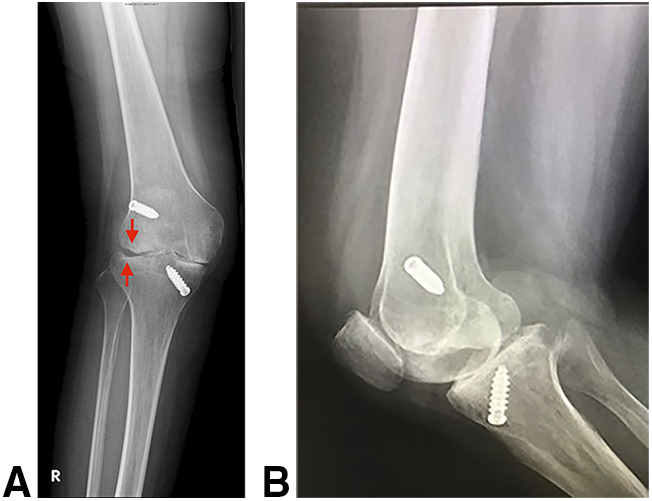
Preoperative radiograph of the right knee showed bone loss over the lateral femoral condyle and lateral tibial plateau depression fracture (**A**) and posterior migration of the tibia (**B**)

**Figure 2:**
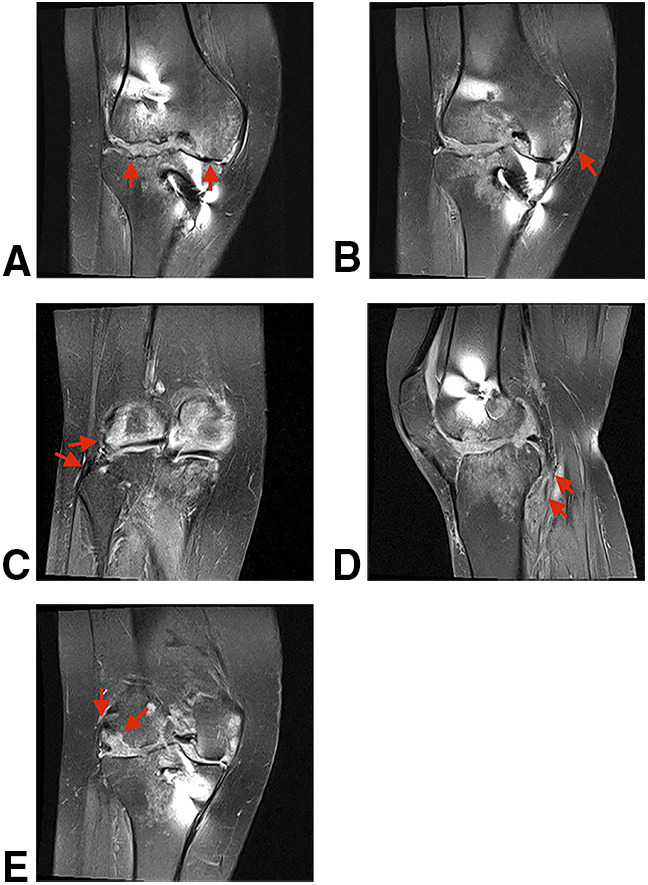
Preoperative MRI of the right knee showed extensive medial and lateral meniscus loss (**A**), decreased thickness of the MCL (**B**), LCL partial tear (**C**), popliteus muscle atrophy with thinning of the tendon part (**D**), extensive chondral loss with cortical erosions in both femoral condyles, and an occult lateral tibial plateau fracture (**E**).

Preoperative lower limbs scanogram and right knee radiograph showed 17° of valgus deformity, a laterally deviated mechanical axis of 6.2 cm, 73.5° of lateral distal femur angle, and bone loss over the lateral femoral condyle and the lateral tibial plateau (Figure 1). MRI showed extensive medial and lateral meniscus loss, decreased thickness of the medial collateral ligament (MCL), an lateral collateral ligament (LCL) partial tear, popliteus muscle atrophy with thinning of the tendon part, extensive chondral loss with cortical erosions in both femoral condyles, and an occult lateral tibial plateau fracture (Figure 2).

### Surgical Technique

**Figure 3:**
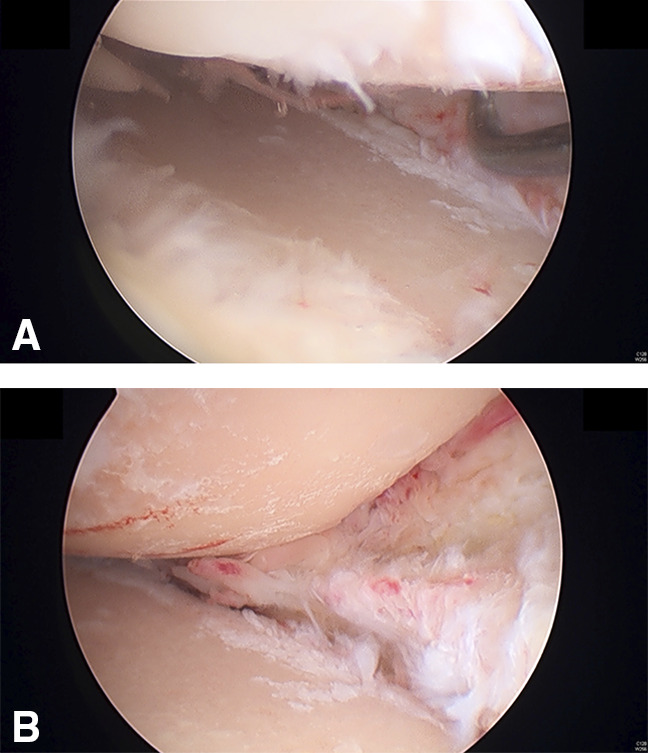
Arthroscopy of the right knee showed extensive osteoarthritic changes in both the medial and lateral femoral condyle and tibial plateau and total loss of the medial and lateral meniscus (**A**, **B**).

The patient underwent right knee arthroscopic meniscus debridement, followed by distal femoral varus osteotomy and PLC reconstructions by using the LaPrade technique^8^ using ipsilateral semitendinosus and gracilis tendon autografts. Arthroscopy showed grade 4 posttraumatic degenerative change in the lateral femoral condyle and tibial plateau, grade 3 posttraumatic degenerative change in the medial femoral condyle and tibial plateau, and a total loss of the medial and lateral menisci (Figure 3). The artificial anterior cruciate ligament (ACL) graft was firm and stable.

**Figure 4:**
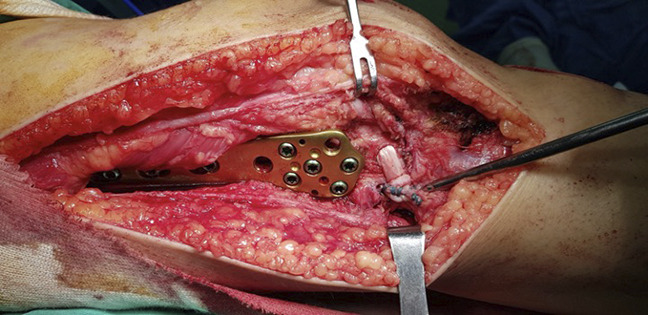
Intraoperative image of the lateral distal femoral osteotomy and posterolateral complex reconstruction.

First, the loose bodies were excised, and the ruptured meniscus remnants were débrided with a motorized shaver. Microfractures were made on the femoral condyles and the tibial plateau. Second, distal femoral osteotomy (DFO) with a varus correction angle of 15° was performed with an Arbeitsgemeinschaft Fur Osteosynthesefragen Less Invasive Stabilization System (AO LISS) plate. The osteotomy site was grafted with an allogeneic femoral head. Finally, the popliteus tendon, LCL, and popliteofibular ligament were reconstructed with semitendinosus and gracilis autografts harvested from the ipsilateral leg (Figure 4). Varus and valgus stress were exerted postoperatively, and the knee was stable.

### Postoperative Rehabilitation

The goal was to decrease pain, inflammation, and swelling; to reestablish quadriceps control; and to restore a normal gait. After the surgical procedure, a fully extended functional knee brace was applied for the first postoperative week. Ambulation with partial weight bearing on the surgical leg was initiated on the second postoperative day. Quadriceps isometric exercises, straight-leg raising exercises, and passive range of motion should be initiated as early as possible. The protected range of motion was gradually increased from 0° to 90° during the first postoperative month and then from 90° to 120° during the second month. At 4 weeks, a series of closed kinetic chain exercises were demonstrated to the patients, who were strongly encouraged to exhibit cogent participation. At 6 weeks, patients were allowed full weight bearing with hamstring-strengthening exercises. The brace was unlocked, and the patient was advised to establish a normal gait and a passive range of motion. At 8 weeks, the active range of motion in the knee should progress to complete flexion and extension. Quadriceps and hamstring muscle strength training was especially emphasized during a home rehabilitation program. At 3 months, patients usually returned to their normal daily activity and were allowed to exercise on a stationary bike or stand on only the repaired leg. Light sport activities began at 6 months. After 9 months, full activity, including athletic activities, was permitted.

### Postoperative Follow-up

**Figure 5:**
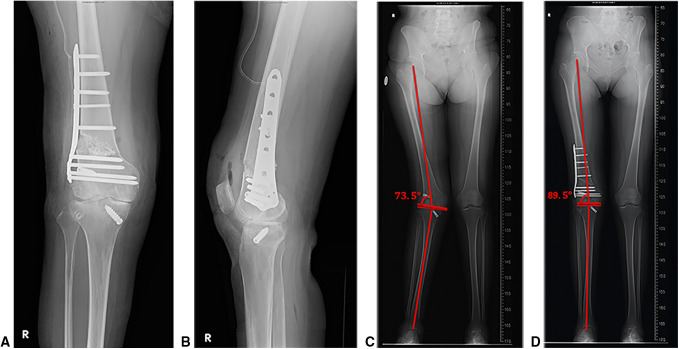
Postoperative knee radiograph (**A**, **B**) and a comparison of the lower limb scanogram (**C**, **D**).

The preoperative mechanical tibiofemoral angle was 17° valgus, and it improved to 5° valgus at the final follow-up. The preoperative mechanical lateral distal femoral angle was 73.5°, and it improved to 89.5° at the last follow-up (Figure 5). The Lysholm score and International Knee Documentation Committee (IKDC) were 52 and 45 preoperatively, and they improved to 87 and 89, respectively, at the 24-month follow-up postoperatively. Currently, the patient does not have symptoms from posterior instability, and the physical examination showed grade 1 posterior laxity with a relatively stable knee and good functional recovery. There was no need for a second-stage posterior cruciate ligament (PCL) reconstruction for the patient.

## Discussion

To the best of our knowledge, this is the first case report of posttraumatic MLKIs treated by allograft anterior cruciate ligament reconstruction only and presented with valgus malalignment and chronic PLC disruption because of residual instability postoperatively.

The genu valgum was determined according to the malalignment test described by Paley et al.^9^ According to the obtained measurement, the patient was assigned to the classification of condylar malalignment owing to depressed lateral tibial plateau and lateral femoral condyle. To address the intra-articular defect, Paley^10^ was the first to mention intra-articular osteotomy for the treatment of valgus deformity and instability secondary to lateral femoral condylar hypoplasia. Feldman et al^11^ reported a retrospective report of five patients who had lateral femoral condylar hypoplasia with or without concomitant lateral hemiplateau depression treated by intra-articular osteotomy. Intra-articular osteotomy provided satisfactory correction of the genu valgum without the creation of an oblique joint line.^11^ However, in this case, a bone tunnel for PLC reconstruction would require an intact lateral femoral condyle, tibial plateau, and fibular head. Intra-articular osteotomy would violate the passage of the bone tunnel needed for the LaPrade procedure.^12^ Therefore, either varus-producing high tibial osteotomy or DFO were considered. Kerkhoffs et al^13^ reported a case series of 23 patients with painful posttraumatic lateral depression and valgus malunion of the proximal part of the tibia treated by proximal tibial varus osteotomy. Twenty of 27 (87%) patients showed good to excellent subjective results after five years of postoperative follow-up. High tibial varus-producing osteotomy can provide satisfactory long-term results in active patients with painful and disabling posttraumatic lateral depression and valgus malunion of the proximal part of the tibia.

In this case, the preoperative valgus angle was 17° and the mechanical lateral distal femoral angle was 73.5°, which indicated that the malalignment was mainly from femoral condylar depression. It is more logical to make a correction with a varus-producing DFO than an osteotomy on the morphologically normal tibial side. In addition, Coventry^14^ suggested that varus-producing DFO is preferable in patients whose valgus angulation exceeds 12° over closing wedge varus osteotomy of the proximal part of the tibia. Finally, we chose to correct the alignment with varus-producing DFO. The patient's postoperative scanogram returned to 5° valgus angulation postoperatively without presentation of joint line obliquity.

In patients with chronic MLKIs who failed reconstruction, recent evidence suggests that both maintaining joint alignment and ligamentous stability are equally important.^15^ The knee, in this case, became stable after addressing the alignment and PLC reconstruction, but slight posterior sagging still persisted despite a normal PCL. We decided to adopt a staged surgery for PCL reconstruction if posterior translation of more than 10 mm during posterior drawer test or isolated grade III PCL tears confirmed by MRI despite nonsurgical management.^16^ Although one-stage reconstruction may seem ideal for correcting all components of instability, the possibility of arthrofibrosis should be considered cautiously. Fanelli et al^8^ suggested that delaying reconstruction for 2 to 3 weeks may decrease the incidence of arthrofibrosis. In addition, this is a salvage surgery, and the creation of a normal coronal alignment while avoiding any possible risks of arthrofibrosis and starting an immediate rehabilitation program is of paramount importance.

Vicenti et al^17^ reported a meta-analysis of four studies comparing repair and reconstruction in MLKIs. A higher failure rate (39% versus 8%) and a lower return-to-sport rate (25% versus 51%) were found in patients who underwent the repair of the PLC compared with reconstruction. In the setting of combined injury to both ACL and PLC, failure to identify and address PLC injury was highlighted as a key factor in ACL reconstruction failure.^18,19^ A meta-analysis result from Bonanzinga et al^20^ suggested reconstruction of both ACL and PLC provided better objective functional outcome than ACL reconstruction with PLC managed either nonsurgically or direct repaired. Therefore, anatomic reconstruction of PLC with the LaPrade technique was chosen rather than direct repair.

The revision of ACL reconstruction remains challenging for both patients and surgeon. Luckily, previous allograft did not stretch out in this case. If allograft failure should happen, choose upon autograft for revision was 2.78 times less likely to sustain a subsequent graft rupture compared with allograft.^21^ Quadriceps tendon and bone-patellar tendon-bone remained optional in this case. Mouarbes et al^22^ reported a meta-analysis suggesting that quadriceps tendon was a better choice compared with bone-patellar tendon-bone and hamstring-tendon because less harvest site pain and better functional outcome was found.

Valgus deformity coexisting with PLC disruption and thinning of the MCL are rare, and no literature detailing the treatment of this type of injury exists. We described our approach to treat a young, active patient who went on to regain excellent function 8 months postoperatively. Our tendency for this kind of injury is to correct the alignment first, combined with ligament reconstruction to restore knee stability during a one-stage procedure. Delaying reconstruction of PCL 3 weeks later may be adopted if grade III PCL avulsion were confirmed by either physical examination or image despite being managed nonsurgically.

## Conclusion

For young patients with MLKIs and valgus malalignment, simultaneous distal femoral varus osteotomy and PLC reconstruction could be the first choice to recover their knee function and prevent the need for joint arthroplasty.

## References

[1] Harner CD Waltrip RL Bennett CH Francis KA Cole B Irrgang JJ: Surgical management of knee dislocations. J Bone Joint Surg Am 2004;86:262-273. 14960670 10.2106/00004623-200402000-00008

[2] Liow RY McNicholas MJ Keating JF Nutton RW: Ligament repair and reconstruction in traumatic dislocation of the knee. J Bone Joint Surg Br 2003;85:845-851. 12931803

[3] Moatshe G Chahla J LaPrade RF Engebretsen L: Diagnosis and treatment of multiligament knee injury: State of the art. J ISAKOS 2017;2:152-161.

[4] Roessler PP Getgood A: The role of osteotomy in chronic valgus instability and hyperextension valgus thrust (medial closing wedge distal femoral varus osteotomy and lateral opening wedge high tibial osteotomy). Clin Sports Med 2019;38:435-449. 31079773 10.1016/j.csm.2019.02.012

[5] Arthur A LaPrade RF Agel J: Proximal tibial opening wedge osteotomy as the initial treatment for chronic posterolateral corner deficiency in the varus knee: A prospective clinical study. Am J Sports Med 2007;35:1844-1850. 17724096 10.1177/0363546507304717

[6] Franciozi CE Albertoni LJB Gracitelli GC: Anatomic posterolateral corner reconstruction with autografts. Arthrosc Tech 2018;7:e89-e95. 29593980 10.1016/j.eats.2017.08.053 PMC5869793

[7] Helito CP Sobrado MF Giglio PN: Posterolateral reconstruction combined with one-stage tibial valgus osteotomy: Technical considerations and functional results. Knee 2019;26:500-507. 30635152 10.1016/j.knee.2018.12.001

[8] Fanelli GC Edson CJ Reinheimer KN: Evaluation and treatment of the multiligament-injured knee. Instr Course Lect 2009;58:389-395. 19385550

[9] Paley D: Principles of Deformity Correction. Berlin: Springer, 2005.

[10] Paley D: Intra-articular osteotomies of the hip, knee, and ankle. Oper Tech Orthop 2011;21:184-196.

[11] Feldman DS Goldstein RY Kurland AM Sheikh Taha AM: Intra-articular osteotomy for genu valgum in the knee with a lateral compartment deficiency. J Bone Joint Surg Am 2016;98:100-107. 26791030 10.2106/JBJS.O.00308

[12] Serra Cruz R Mitchell JJ Dean CS Chahla J Moatshe G LaPrade RF: Anatomic posterolateral corner reconstruction. Arthrosc Tech 2016;5:e563-572. 27656379 10.1016/j.eats.2016.02.006 PMC5021087

[13] Kerkhoffs GM Rademakers MV Altena M Marti RK: Combined intra-articular and varus opening wedge osteotomy for lateral depression and valgus malunion of the proximal part of the tibia. J Bone Joint Surg Am 2008;90:1252-1257. 18519318 10.2106/JBJS.D.01816

[14] Coventry MB: Proximal tibial varus osteotomy for osteoarthritis of the lateral compartment of the knee. J Bone Joint Surg Am 1987;69:32-38. 3805069

[15] Phisitkul P Wolf BR Amendola A: Role of high tibial and distal femoral osteotomies in the treatment of lateral-posterolateral and medial instabilities of the knee. Sports Med Arthrosc Rev 2006;14:96-104. 17135954 10.1097/01.jsa.0000212306.47323.83

[16] Montgomery SR Johnson JS McAllister DR Petrigliano FA: Surgical management of PCL injuries: Indications, techniques, and outcomes. Curr Rev Musculoskelet Med 2013;6:115-123. 23430587 10.1007/s12178-013-9162-2 PMC3702782

[17] Vicenti G Solarino G Carrozzo M: Major concern in the multiligament-injured knee treatment: A systematic review. Injury 2019;50(suppl 2):S89-s94. 30797544 10.1016/j.injury.2019.01.052

[18] Corten K Bellemans J: Cartilage damage determines intermediate outcome in the late multiple ligament and posterolateral corner-reconstructed knee: A 5- to 10-year follow-up study. Am J Sports Med 2008;36:267-275. 18182652 10.1177/0363546507311091

[19] Ménétrey J Duthon VB Laumonier T Fritschy D: “Biological failure” of the anterior cruciate ligament graft. Knee Surg Sports Traumatol Arthrosc 2008;16:224-231. 18183368 10.1007/s00167-007-0474-x

[20] Bonanzinga T Zaffagnini S Grassi A Marcheggiani Muccioli GM Neri MP Marcacci M: Management of combined anterior cruciate ligament-posterolateral corner tears: A systematic review. Am J Sports Med 2014;42:1496-1503. 24220017 10.1177/0363546513507555

[21] MARS Group: Effect of graft choice on the outcome of revision anterior cruciate ligament reconstruction in the Multicenter ACL Revision Study (MARS) Cohort. Am J Sports Med 2014;42:2301-2310. 25274353 10.1177/0363546514549005 PMC4447184

[22] Mouarbes D Menetrey J Marot V Courtot L Berard E Cavaignac E: Anterior cruciate ligament reconstruction: A systematic review and meta-analysis of outcomes for quadriceps tendon autograft versus bone-patellar tendon-bone and hamstring-tendon autografts. Am J Sports Med 2019;47:3531-3540. 30790526 10.1177/0363546518825340

